# Increased Expression of CCN2 in the Red Flashing Light-Induced Myopia in Guinea Pigs

**DOI:** 10.1155/2013/761823

**Published:** 2013-07-14

**Authors:** Hong Wang, Kang Zhuang, Lei Gao, Linna Zhang, Hongling Yang

**Affiliations:** Department of Ophthalmology, Qilu Hospital, Shandong University, 107 Wenhua Xi Road, Jinan 250012, China

## Abstract

Visual environment plays an important role in the occurrence of myopia. We previously showed that the different flashing lights could result in distinct effects on the ocular growth and development of myopia. CCN2 has been reported to regulate various cellular functions and biological processes. However, whether CCN2 signaling was involved in the red flashing light-induced myopia still remains unknown. In the present study, we investigated the effects of the red flashing lights exposure on the refraction and axial length of the eyes *in vivo* and then evaluated their effects on the expression of CCN2 and TGF-**β** in sclera tissues. Our data showed that the eyes exposed to the red flashing light became more myopic with a significant increase of the axial length and decrease of the refraction. Both CCN2 and TGF-**β**, as well as p38 MAPK and PI3K, were highly expressed in the sclera tissues exposed to the red flashing light. Both CCN2 and TGF-**β** were found to have the same gene expression profile *in vivo*. In conclusion, our findings found that CCN2 signaling pathway plays an important role in the red flashing light-induced myopia *in vivo*. Moreover, our study establishes a useful animal model for experimental myopia research.

## 1. Introduction

Myopia is a vision condition in which the visual images come to a focus in front of the retina of the eye because of defects in the refractive media of the eye or of abnormal length of the eyeball, resulting especially in defective vision of distant objects. It has emerged as a significant public health issue in the world because of its increasing prevalence. The prevalence of myopia has been reported as high as 70–90% in Asia, 30–40% in Europe and United States, and 10–20% in Africa [1]. More and more studies revealed that early myopia might affect ocular growth and refractive status. Myopia is still a challenging disease to treat because the mechanisms of the development of myopia and the host response of myopic genes are not yet fully understood. An improved understanding of the effects of vision environment on the development of myopia is urgently required to identify potential preventative and therapeutic strategies. 

 In addition to the well-known cause of myopia genes and bad posture habits, the flashing lights may be directly associated with myopia with the growing popularity of computers and television. More and more studies have shown that many individuals who work at a computer experience high level of eye or vision disorders, such as the visual fatigue [2]. Our previous study revealed that flashing light could cause axial elongation and further induce myopia in pigmented guinea pigs. Moreover, different wavelength of unique flashing lights had distinct influences, and the most effective light is the red light (750 nm) [3]. Therefore, in this study, we will further focus on the effects of the red flashing light on the ocular growth and refraction in guinea pigs. 

 Connective tissue growth factor (CTGF/CCN2) is an extracellular matrix protein composed of four domains which belongs to the CCN protein family. CCN2 has been identified to be involved in various cellular functions and biological processes including fibrosis, angiogenesis, differentiation, and wound healing in various cell types [4–7]. Transforming growth factor-*β* (TGF-*β*) is a multifunctional cytokine involved in diverse cellular processes such as cell proliferation, apoptosis, differentiation, and migration [8, 9]. Its dysfunctions could lead to various kinds of diseases such as tissue fibrosis and cancer [10]. Previous study reported that TGF-*β* functioned as the direct inducer of CCN2 expression *in vitro* and *in vivo* studies [11]. Our previous study revealed that the flashing light could induce the cell number and activity of posterior sclera cells which resulted in an abnormal proliferation status of sclera in guinea pigs [12], indicating that active sclera remodeling plays a significant role in the flashing light-induced ocular growth and vision impairment. However, it is still unknown whether CCN2 and TGF-*β* are also involved in the red flashing light-induced vision change and myopia. The exact role of CCN2 in the progression of experimental myopia has yet to be determined. 

 In the present study, we exposed the guinea pigs under the red flashing light condition and further evaluated its effects on the changes of refractive error and axial length. We also investigated the expression of CCN2 and TGF in sclera tissues of the red flashing light-induced myopia model to explore their role in the pathogenesis of experimental myopia.

## 2. Materials and Methods

### 2.1. Myopic Animal Model

 The animal research procedures in this study were approved by the Animal Care and Ethics Committee at Shandong University School of Medicine. The treatment and care of animals were conducted according to the ARVO statement for the Use of Animals in Ophthalmic and Vision Research. Thirty guinea pigs (aged from 15 to 20 days) weighting from 70 to 90 grams had similar refractive error (from +2.00 to +3.50D) and were obtained at Experimental Animal Center of Shandong University. They were randomly assigned to 3 groups (*n* = 10 each group). Group 1 was in a tightly closed carton and exposed to the red flashing light (see Supplementary Figure S1A available online at http://dx.doi.org/10.1155/2013/761823). Group 2 was in a tightly closed around carton and exposed to the white flashing light (Supplementary Figure S1B). Group 3 was in a sight-widen cage and exposed to the natural light (Supplementary Figure S1C). For exposure to the red flashing light, a PS-I programmable flash stimulator (Supplementary Figure S1D) was performed with 100 red diode and circuit board to produce the red light. All the animals were exposed on a cycle of 12 h illumination (800 lux) and 12 h darkness daily during the experimental period. The 800 lux lamps were turned on and off with a 2 sec-2 sec on-off cycle. The animals were sacrificed at the end of light exposure.

### 2.2. Refraction and Axial Length Measurement

The refraction and axial length of eyes were checked in the guinea pigs at 8 weeks after light exposure. One drop of 0.5% tropicamide was administered to the eyes every 5 min for twice to achieve a completely dilated pupil. After 30 min, retinoscopy was performed (accuracy: 0.25D) in a dark room using a streak retinoscope. The refraction was evaluated by the mean value of the horizontal and vertical meridians. The animal specific A-scan ultrasonography was used for the axial length measurement. Before the ultrasound measurement, 0.5% oxybuprocaine hydrochloride eye drops was administered for twice for topical anesthesia. The ultrasound probe directly contacted with the cornea during the axial length measurement. All the data represented averages from 10 repeated measurements.

### 2.3. Tissue Preparation

 All the animals were euthanized with an overdose of 3% pentobarbital sodium and the eyeballs were enucleated with the sclera immediately. The cornea central horizontal diameter at the nasal and temporal limbus were marked, and then the bulbar conjunctiva along the limbus was cut off to remove the anterior segment of the organization and vitreous washed with saline choroidal pigment residues on the sclera. Part of sclera tissues were then stored in −80°C low temperature refrigerator for further mRNA and protein detection, and another part of sclera tissues were fixed in buffered 4% formalin solution (PH = 7.4) and embedded in paraffin.

### 2.4. Immunohistochemistry Assay

 Slides were dewaxed and rehydrated using xylene and graded alcohols, followed by the incubation in the 3% H_2_O_2_ solution for 10 min to quench the endogenous peroxidise activity. The tissue section was heated by a microwave oven to repair the antigen. Nonspecific binding was blocked with normal goat serum for 30 min. The slides were incubated with the diluted primary antibody (rabbit anti-mouse CCN2 antibody, Boster Bioengineering Limited Company, China) at 4°C overnight. The following morning the biotinylated goat anti-rabbit IgG antibody was added to the tissue section and incubated for 30 min. Next, SABC peroxidase and DAB chromogenic kit were applied. DAB developing time was controlled based on observation under bright-field light microscopy. The tissue sections were slightly counterstained with haematoxylin and then washed with distilled water to clean haematoxylin. Finally, the tissue section was dried by baking and sealed by a drop of resin with a cover slide. PBS was applied as negative control.

### 2.5. Quantitative Real-Time PCR

 Total RNA was extracted with trizol reagent purchased from Shanghai Sangon Biological Engineering Technology Company. Briefly, the concentration and purity were determined by ZF spectrophotometry (Shanghai Kang Huasheng instrument factory). The RNA integrity was verified by electrophoresis in 3% denaturing formaldehyde agarose gel. Subsequently, 1 *μ*g of total RNA was used to synthesize cDNA by SuperScript III kit (Invitrogen) according to the manufacture's instructions. Real-time PCR was performed on SDS 7500 instrument (Applied Biosystems) with the Taq polymerase and primers. Primers of PCR were designed using Primer Express 3.0 software (Applied Biosystems) and synthesized by Shanghai Bioon Biotechnology. The primers were as follows: CCN2, forward: 5′-TCTCCACCCGAGTTACCAATG-3′, reverse: 5′-CACCCCGCAGAACTTAGCC-3′; TGF-*β*, forward: 5′-TTGAACTCAGAGACGTAAGCGT-3′, reverse: 5′-AGCGCCAGGAATTGTTGCT-3′, p38MAPK, forward: 5′-AAGTTCCTGTCCACATTGCC-3′, reverse: 5′-TGGATTCAGTGTCAAGCTGC-3′; PI3 K, forward: 5′-CGAGAGTGTCGTCACAGTGTC-3′, reverse: 5′-TGTTCGCTTCCACAAACACAG-3′; *β*-Actin, forward: 5′-CTGTTGCTCGCGTCGCTATA-3′, reverse: 5′-AACGATGCCGTGCTCAATG-3′. The amplification process was as follows: predenaturation for 5 min at 95°C, followed by 40 cycles of denaturation at 95°C for 15 s, annealing at 60°C for 30 s, extension at 72°C for 30 s, and a final extension at 72°C for 5 min. Fluorescence reading was taken during 60°C step. Equal volume of PCR products was electrophorezed and photographed under ultraviolet illumination. The relative mRNA expression level of each gene was calculated with the ΔΔ*C*_*t*_ method. *β*-Actin was used as control in the same sample.

### 2.6. Western Blot Analysis

 The sclera tissues were homogenized and lysed with ice-cold radioimmunoprecipitation (RIPA) buffer containing protease inhibitors. Protein concentration was then quantified by Bradford Protein Assay Kit. Equal amounts of protein were boiled, loaded on a 10% SDS-PAGE gel, and transferred onto a nitrocellulose membrane. The membrane was blocked with 5% nonfat dry milk in tris-buffered saline containing 0.1% Tween20 (TBST) for 1 h, exposed to rabbit anti-mouse CCN2 antibody (Boster Bio-engineering Limited Company, China), and incubated overnight at 4°C. The same blots were stripped and reanalyzed using anti-Actin antibody as the internal control. Membranes were washed with TBST and then incubated with the horseradish-peroxidase- (HRP-) labeled antibody for 1 h. The protein bands were visualized using Immobilon Western Chemiluminescent HRP Substrate reagents and exposed to autoradiography film, developed, and fixed. The film was scanned and analyzed with Quantity One Analyzer Software. Independent experiments were repeated three times.

### 2.7. Statistical Analysis

 All data were expressed as the mean ± SEM from at least three independent experiments. Statistical comparisons between the groups, including corneal radius of curvature, eye refractive error, axial length, the TGF-*β*, and CCN2 expression levels, were performed by a one-way ANOVA using SPSS (version 11.0). Statistical comparisons among CCN2 immunohistochemical positive rates were performed by chi-square test. *P* < 0.05 was considered to be statistically significant. 

## 3. Results 

### 3.1. Confirmation of Phenotypic Changes Induced by Red Flashing Light

**Figure 1:**
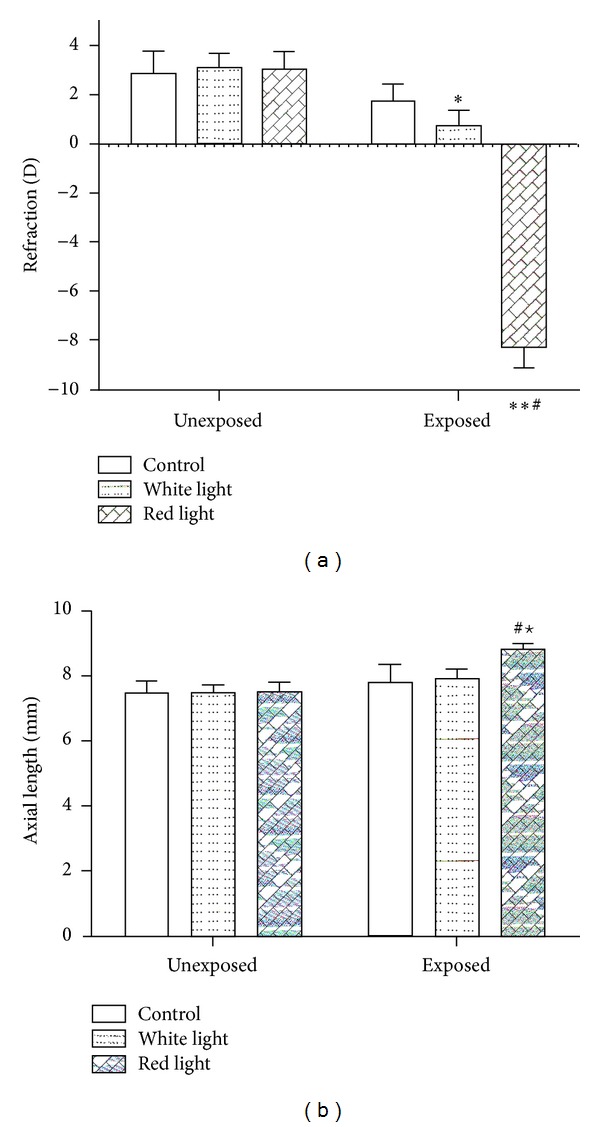
Effects of red flashing light on refraction and axial length in guinea pigs. (a) The refraction was detected using a streak retinoscope. (b) The axial length was evaluated by A-scan ultrasonography. Data shown are from three independent experiments. *  and ^⋆^ *P* < 0.05 indicate significant differences from unexposed groups; ^#^ *P* < 0.05 indicates significant difference from white flashing light group.

 There was no significant difference of refraction and axial length between the two eyes of the same animal at the beginning of the experiment. The eyes after red flashing light exposure became more myopic by −11.29 D (Figure 1(a)) and had an increase of axial length by 1.32 mm (Figure 1(b)). In white flashing light exposure group, the refraction was also clearly decreased by +2.35 D (Figure 1(a)) with an elongation of axial length by 0.41 mm (Figure 1(b)) compared to the eyes under natural light exposure (Figures 1(a) and 1(b)). Interestingly, the refraction and axial length data showed that the natural light control eyes also had a myopic shift.

### 3.2. CCN2 Protein Expression Was Enhanced in Posterior Sclera after Red Flashing Light Exposure

**Figure 2:**
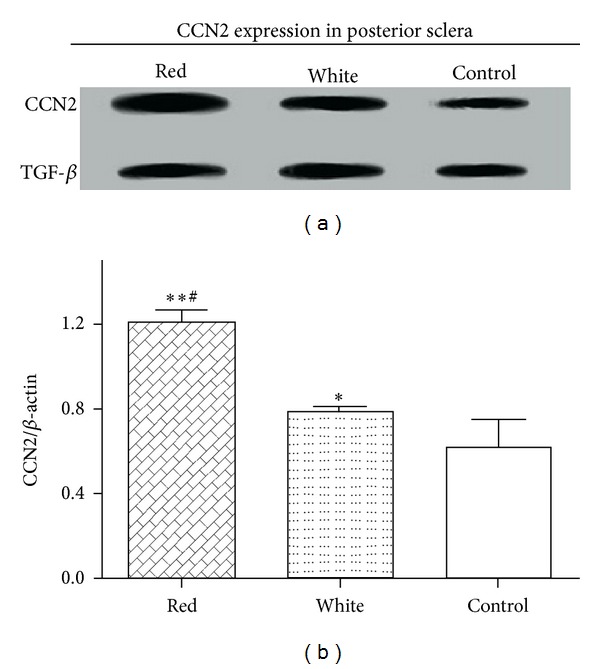
CCN2 protein expression in posterior sclera tissues after light exposure. (a) The protein level of CCN2 was detected by Western blot analysis. (b) The relative CCN2/*β*-actin protein level was evaluated in posterior sclera tissues. *P < 0.05 indicates significant differences from control; **P < 0.01 indicates significant difference from control. ^#^ *P* < 0.01 indicates significant difference from white light group.

 To determine the effect of flashing light exposure on CCN2 expression, the protein level of CCN2 was measured by Western blot analysis. The data showed that the protein levels of CCN2 were the highest in the red flashing light irradiated eyes, followed by the eyes of the white flashing light exposure group and the lowest in the eyes of the natural light controls (Figure 2(a)). The differences of the protein levels between any two groups are shown as in Figure 2(b), and the protein levels in red flashing light exposure group were remarkably increased by 2.0-fold and by 1.3-fold in the white flashing light exposure group. 

### 3.3. CCN2 Immunostaining Was Increased after Flashing Light Exposure

**Figure 3:**
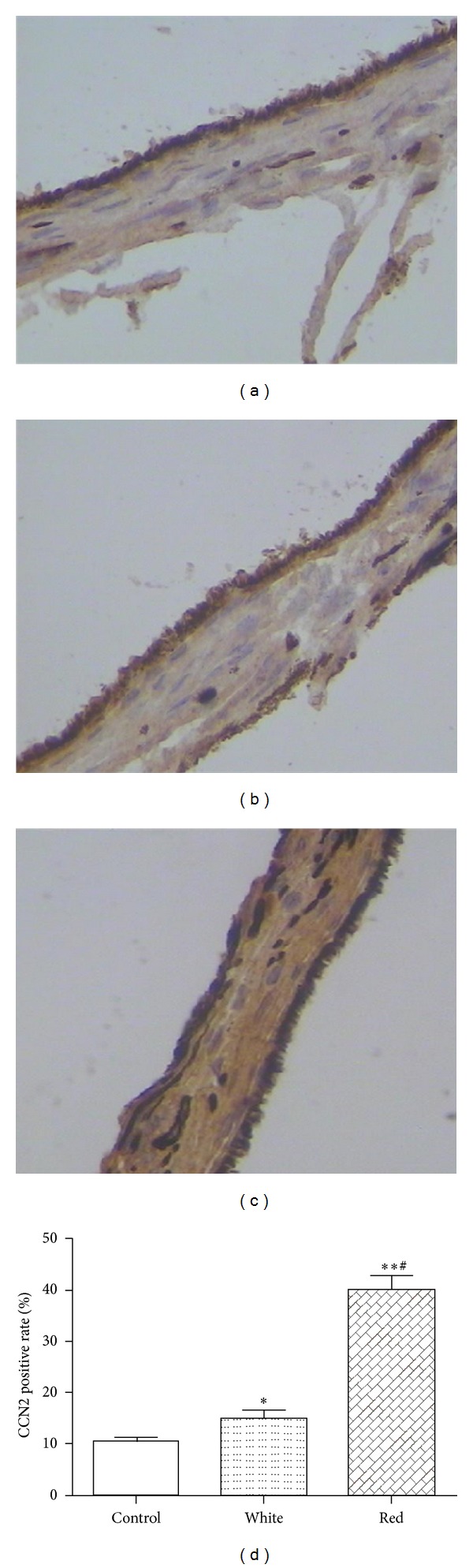
CCN2 immunostaining in posterior sclera tissues after light exposure. ((a)–(c)) CCN2 expression was stained in control, white light and red light exposed sclera tissues, respectively. (d) CCN2 positive rate in posterior sclera tissues after light exposure. *P < 0.05 indicates significant differences from control; **P < 0.01 indicates significant difference from control; ^#^ *P* < 0.01 indicates significant difference from white light group.

 To further confirm the effect of flashing light exposure on CCN2 expression, the levels of CCN2 in the sclera tissues of guinea pigs exposed to the natural light, white flashing light, and red flashing light were compared with immunohistochemistry. The data showed that the level of CCN2 immunostaining was detectable in control sclera tissues (Figure 3(a)), increased in white flashing light group (Figure 3(b)), and significantly further increased in the red flashing light group (Figure 3(c)). The CCN2 positive rate was (10.36 ± 0.78) %, (15.04 ± 0.59) %, and (40.21 ± 0.65) % in the eyes of natural light, white light, and red light exposure groups, respectively (Figure 3(d)). These data indicated that the red flashing light exposure could significantly induce CCN2 expression in sclera tissue of guinea pigs.

### 3.4. Both TGF-*β* and CCN2 Were Overexpressed in Flashing Light-Induced Myopia Model

**Figure 4:**
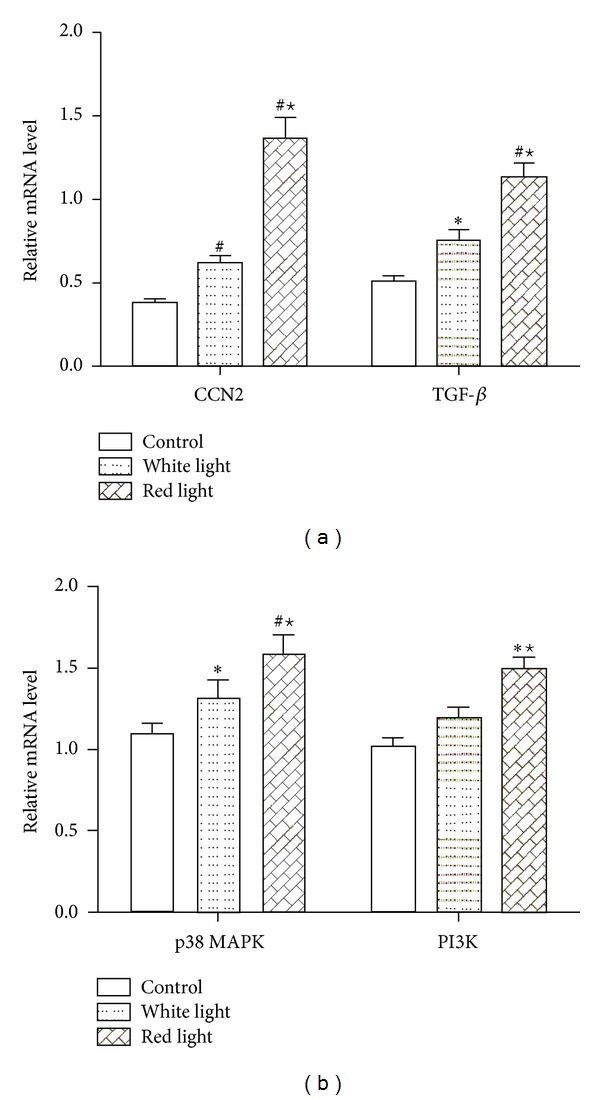
The expression of CCN2 and downstream pathway genes in posterior sclera tissues after light exposure was evaluated by RT-PCR. (a) The relative CCN2 and TGF-*β* mRNA level were analyzed in posterior sclera tissues. (b) The relative p38 MAPK and PI3K mRNA level were also examined in posterior sclera tissues. *P < 0.05 indicates significant differences from control; ^#^ *P* < 0.01 indicates significant difference from control; ^⋆^ *P* < 0.05 indicates significant differences from control.

As CCN2 is a direct target gene of TGF-*β* signaling, the contribution of TGF-*β* in red flashing light-induced CCN2 expression was analyzed in sclera tissues. The mRNA levels of TGF-*β*, and CCN2 were detected by RT-PCR. The data demonstrated that the expression of TGF mRNA in both the red light and white light exposed eyes increased compared to the natural light exposed eyes, and such an increase in the red flashing light exposed eyes was more obvious than in white flashing light exposed eyes (Figures 4(a) and 4(b)). The expression of CCN2 mRNA was lower in natural light control eyes but increased in the white light exposed eyes and more apparently increased in the red flashing light exposed eyes. These data indicated that both TGF and CCN2 were coexpressed in sclera tissues in flashing light-induced myopia model, suggesting that TGF-*β*-CCN2 signaling might play an important role in the ocular growth and development of myopia.

## 4. Discussion

 In this study, we used the flashing light to induce myopia in the guinea pigs and compared the refraction and axial length among the red flashing light and white flashing light exposed eyes of the experimental animals and natural light control animals. The eyes under red flashing light exposure became more myopic and had an obvious increase of axial length, suggesting that flashing light is related to ocular growth and further development of myopia. This result establishes a general and useful animal model for experimental myopia research. We further compared CCN2 and TGF-*β* expression levels in sclera among the experimental and control animals. The eyes exposed to red flashing light had the highest expression level, followed by the eyes exposed to white flashing light, and eyes of natural light control animals had the lowest expression levels. We also noticed that both CCN2 and TGF-*β* have the same gene expression profile in sclera tissues of myopic model. These data indicated that TGF-*β*-CCN2 signaling might play an important role in the development of myopia. From the CCN2 and TGF-*β* expression data and the change of refraction and axial length, the red flashing light could clearly promote ocular growth and induce myopia. We think that the red flashing light might induce myopia through the emmetropization interruption in both eyes of animals and the physiologic control of gene expression.

 Guinea pigs have been increasingly considered to be an alternative model in the study of experimental myopia since the biometric changes of guinea pig eye are similar to those of other species and are relatively susceptible to develop myopia [13–15]. Moreover, they are very cooperative, less expensive, easily handled, readily responded [16, 17], and born with a well-developed vision system [18] than most of other small mammals. In this study, the red flashing light-induced myopic animal model completely meets four basic requirements of myopic animal models. Firstly, it strictly follows the replacement of the 12 h circadian rhythm. The red flashing light has a similar intensity of 800 lux with the normal natural light. The visual environment alters between light and dark during red light exposure, and the image on the retina is constantly changing from clear to blurred. Secondly, it maintains the integrity of the retinal anatomic structures. There was no toxicity to the eyes exposed to the red flashing light intensity of 800 lux in this experiment. Thirdly, the starting time of the experiment begins at the visual sensitive period of the guinea pig. All the animals that aged from 15 to 20 days began to accept the light stimulation at the sensitive period. Finally, it maintains the continuity of the experiment. In this study, all the guinea pigs were continuously exposed to red flashing light for eight weeks. Therefore, our study for the first time verified that red flashing light-induced myopia in guinea pig represents an important animal model to study ocular growth and myopic development. 

 Human sclera has been considered as a dynamic tissue that can alter extracellular matrix (ECM) components and their biomechanical characters in response to changes in the visual environment to regulate ocular refraction and axial length [19]. It has been reported that active sclera ECM remodeling could result in the development of myopia [20]. Our previous study found that flashing light could lead to an increase of sclera cells number and its biological activity in guinea pigs [12], suggesting that the sclera remodeling was associated with flashing light-induced myopia. However, we still have relatively little understanding of the cellular signaling factors that drive the changes of ocular growth and myopia development.

 CCN2 was first identified as a profibrotic mediator which was upregulated both *in vitro* and *in vivo* models of diabetic nephropathy [21]. More and more studies revealed that CCN2 largely functions as a matricellular protein, regulating and integrating the role of other growth factor signaling, cell motility, and differentiation in tissue remodeling [22]. CCN2 can bind to multiple receptors and activate divergent signaling pathways, including TGF-*β*, p38 MAPK [23], PI3K [24], and Rho GTPase [25]. CCN2 is expressed in a variety of cell types including fibroblasts, endothelial cells, vascular smooth muscle cells, and epithelial cells [26–28]. Previous study reported that the expression of CCN2 was lower in sclera fibroblasts than in corneal fibroblasts, and the sclera appeared normal in the eyes of CCN2 knockout mouse [29]. Our study for the first time found that CCN2, as well as its downstream signaling pathway genes including p38 MAPK and PI3K, was lower in sclera tissue in guinea pigs but increased after white flashing light exposure and significantly increased under red flashing light exposure, suggesting that CCN2 might play an important role in sclera remodeling in the development of myopia. However, to what extent CCN2 gene of small effect and gene-environment interactions contribute to the development of myopia remains to be elucidated.

 In conclusion, the refractive and axial length data indicated that the red flashing light could successfully induce myopia in guinea pigs, providing a useful animal model for the study of experimental myopia. We also identified a significant change of CCN2, TGF-*β*, p38 MAPK, and PI3K expression levels in the eyes of the guinea pigs exposed to the red flashing light. CCN2 signaling pathway might play a significant role in flashing light-induced ocular growth and myopic development. Exploration of CCN2 signaling pathways will help us reveal the molecular mechanisms underlying sclera remodeling and ocular growth.

## Supplementary Material

Supplemental Figure 1: The myopia animal model was constructed under different types of lights exposure. (A-C) The guinea pigs were exposed to the red flashing light (Group 1), white flashing light (Group 2) and natural white light (Group 3), respectively. (D) The guinea pigs were in tightly closed paper cartons and exposed to the flashing lights. From left to right, the carton was exposed to the white, yellow, green and red flashing light emitted by a PS-I programmable flash stimulator. (E) PS-I programmable flash stimulator was provided by Qilu Hospital, Shandong University. It has hundreds of diodes and circuit boards to produce various flashing lights, including white, yellow, green and red. The output power was adjusted appropriately to control the flashing light intensity at 8001ux and the radiation law was on for 2 seconds, dark 2 seconds.

## Abbreviations

CCN2: Connective tissue growth factor
TGF-*β*: Transforming growth factor-*β*
RIPA: Radioimmunoprecipitation
RT-PCR: Reverse transcription-polymerase chain reaction
HRP: Horseradish peroxidase.
