# Supplementary material for: Increased Expression of CCN2 in the Red Flashing Light-Induced Myopia in Guinea Pigs
Source: Biomed Res Int. 2013 Jul 14;2013:761823. doi: 10.1155/2013/761823 (PMC3726013; doi:10.1155/2013/761823)
Supplement: Supplementary file 1 — Supplemental Figure 1: The myopia animal model was constructed under different types of lights exposure. (A-C) The guinea pigs were exposed to the red flashing light (Group 1), white flashing light (Group 2) and natural white light (Group 3), respectively. (D) The guinea pigs were in tightly closed paper cartons and exposed to the flashing lights. From left to right, the carton was exposed to the white, yellow, green and red flashing light emitted by a PS-I programmable flash stimulator. (E) PS-I programmable flash stimulator was provided by Qilu Hospital, Shandong University. It has hundreds of diodes and circuit boards to produce various flashing lights, including white, yellow, green and red. The output power was adjusted appropriately to control the flashing light intensity at 8001ux and the radiation law was on for 2 seconds, dark 2 seconds. [file 761823.f1.docx]

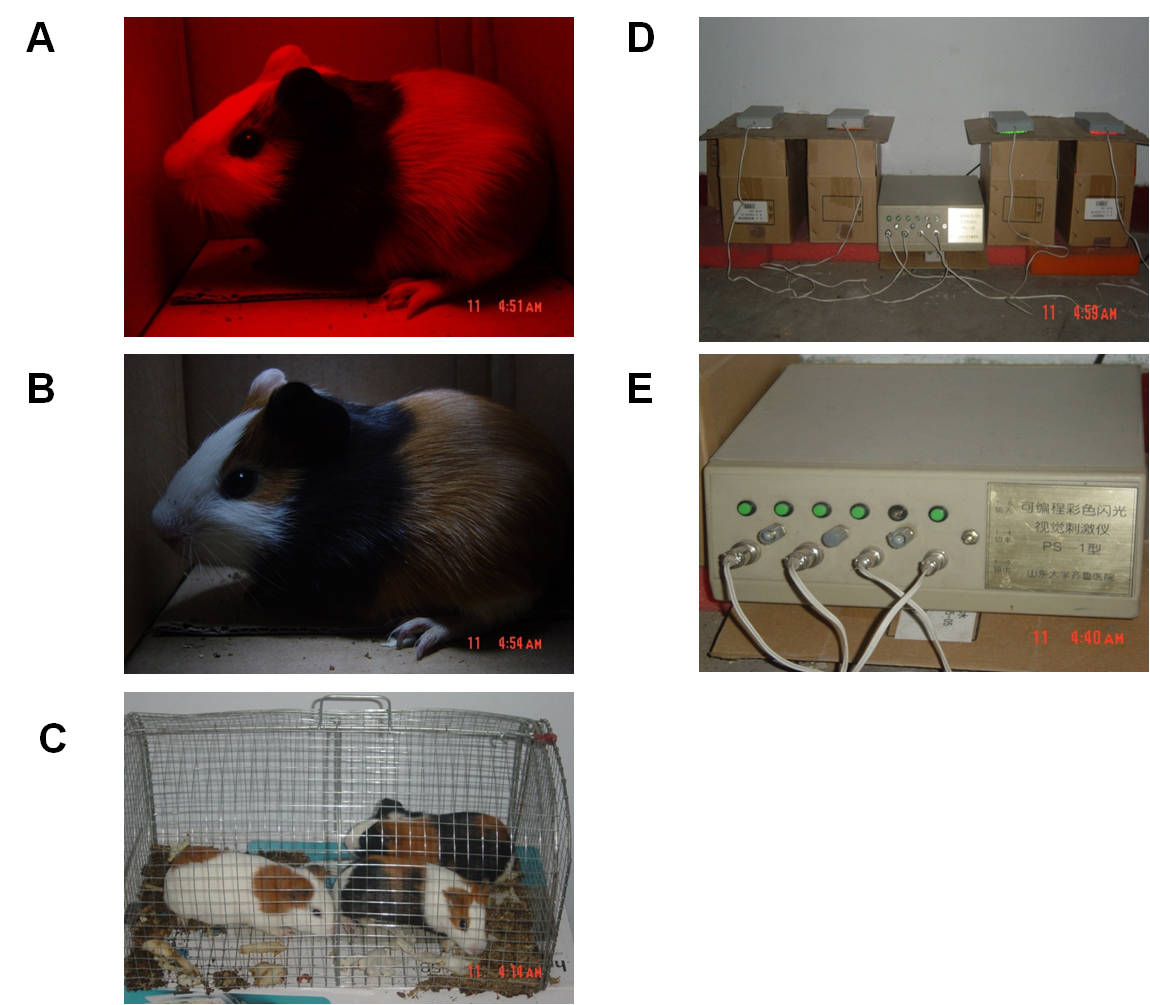


**Fig.S1** The myopia animal model was constructed under different types of lights exposure. (A-C) The guinea pigs were exposed to red flashing light (Group 1), white flashing light (Group 2) and natural white light (Group 3), respectively.(D) The guinea pigs were in tightly closed cartons and exposed to the flashing lights. From left to right, the carton was exposed to the white, yellow, green and red flashing light emitted by a PS-I programmable flash stimulator. (E) PS-I programmable flash stimulator was provided by Qilu Hospital, Shandong University. It has hundreds of diodes and circuit boards to produce various flashing lights, including white, yellow, green and red. The output power was adjusted appropriately to control the flashing light intensity at 8001ux and the radiation law was on for 2 seconds, dark 2 seconds.
